# Biomedical Applications of Collagen

**DOI:** 10.3390/bioengineering10010090

**Published:** 2023-01-10

**Authors:** Ngan F. Huang, Tatiana S. Zaitseva, Michael V. Paukshto

**Affiliations:** 1 Department of Cardiothoracic Surgery, Stanford University, Stanford, CA 94305, USA; 2 Stanford Cardiovascular Institute, Stanford University, Stanford, CA 94305, USA; 3 Center for Tissue Regeneration, Repair and Restoration, Veterans Affairs Palo Alto Health Care System, Palo Alto, CA 94304, USA; 4 Department of Chemical Engineering, Stanford University, Stanford, CA 94305, USA; 5 Fibralign Corporation, Union City, CA 94587, USA

Extracellular matrix proteins (ECMs) provide structural support and dynamic signaling cues that regulate cell behavior and tissue morphogenesis. Among the ECMs in the body, collagen is one of the most abundant ones. Collagen provides mechanical integrity to biological tissues that sustains three-dimensional (3D) shape and stability. On the cellular side, collagen triggers mechanotransduction pathways that regulate cellular processes such as proliferation, differentiation, angiogenesis, and migration [1,2,3]. Owing to the importance of collagen within biological tissues, disruption to collagen deposition and remodeling is associated with aging and disease. For these reasons, therapies that target collagen hold potential for regenerative medicine and tissue engineering. We have previously applied collagen for the treatment of multiple diseases and injuries, including cardiovascular diseases [4,5,6,7], musculoskeletal injury [8,9,10], and lymphedema [11,12,13]. Collagen-based products are actively being used in clinical trials for various biomedical applications, including muscle loss [14], lymphatic regeneration [15], and treatment of chronic ulcers [16,17].

In this editorial, we have assembled comprehensive reviews and original research articles on the state-of-the-art advances in collagen research from basic and translational applications, based on a series of articles published in *Bioengineering* during 2020 and 2021. This collection brings together experts in collagen biophysics, manufacturing, and applications in biomedicine. These applications span the fields of wound healing and skin regeneration, tumor formation, diseases of the airway, and cardiovascular disease. 

One focus area of this collection is the biomanufacturing of collagen and collagen mimetics. Chan et al. describe the application of collagen as a bioink using additive manufacturing techniques, such as extrusion, inkjet printing, laser-assisting printing, and stereolithography printing [18]. Advantages of three-dimensional (3D) bioprinting strategies include the control of scaffold geometry, scalability, and the incorporation of cells into bioinks for tissue engineering [19]. Despite the progress of additive manufacturing, challenges in collagen biomanufacturing remain and they are discussed by the authors. 

Owing to its relevance to many organ systems, including systems characterized by anisotropic cellular organization, collagen can be fabricated with geometric patterning cues. Blackstone et al. review the techniques for generating spatially patterned collagen for tissue engineering applications using electrospinning technology [20]. Electrospun collagen scaffolds are generated with the assistance of an electric field to create continuous strands of fibers that can be deposited with randomly oriented or spatially patterned configurations. Electrospun scaffolds can be generated with a variety of spatial parameters to mimic the native ECM, including fibril diameters, crosslinking degrees, and porosities. The electrospun scaffolds can be further crosslinked to support mechanical strength and resistance to degradation while being permissive to cell survival. The authors discuss the applications of electrospun scaffolds for cardiovascular, wound healing, neural, and musculoskeletal applications. 

In addition to electrospinning, fibrillar scaffolds can also be fabricated by other technologies, including a shear-mediated deposition process [21,22,23], where fibril diameters can be controlled by the ionic strength of the collagen monomeric solution [24]. Another spatial patterning technique involves soft lithography for the fabrication of microgrooved collagen substrates. Kagayama shows that micropatterning of vascular smooth muscle cells induces smooth muscle differentiation while reducing cell proliferation, in part through YAP signaling and nuclear elongation [25]. Together, these various fabrication strategies enable the development of 3D collagen scaffolds for various biomedical applications. 

In addition to the fabrication of scaffolds composed only of collagen, numerous strategies have also been developed to engineer hybrid collagen scaffolds to improve its mechanical or chemical properties or to modulate cell behavior. Walimbe et al. review the benefits of incorporating glycosaminoglycans into collagen hydrogels to regulate cellular processes such as cellular infiltration, differentiation, and angiogenesis [26]. The glycosaminoglycans of interest include hyaluronic acid, chondroitin sulfate, heparin, and alginate. Additionally, Patil et al. review the current strategies to engineer hybrid scaffolds by combining collagen with biomaterials composed of naturally derived synthetic or inorganic materials [27]. In addition to full-length collagen, collagen-derived peptide sequences, such as DGEA or RGD, can be tethered to other biomaterials. Advancements in collagen fabrication have also extended into the engineering of recombinant collagen and collagen-mimetic peptides. Xu et al. review the advancements in producing heterotrimeric collagen-mimetic peptides that can arrange into ordered molecular assemblies [28]. The authors also describe the production of collagen-mimetic peptides using recombinant systems. This strategy allows them to obtain collagen-mimetic peptides of 30–300 amino acid residues in size, of which some can further form collagen-mimetic fibrils. Fertala et al. describe the applications of recombinant collagen for tissue repair, drug delivery, and the delivery of therapeutics [29]. The potential of recombinant collagen as a replacement therapy for individuals with collagen genetic mutations is further discussed. These reports summarize the state of engineered or recombinant collagen for biomedical applications.

To better understand collagen’s structure–function relationship, San Antonio et al. mapped the fibril interactome of collagen type I [30]. Their model of the collagen fibril incorporates multiple stages of collagen bioprocessing, including fibril assembly and crosslinking. Their interactome is consistent with the dynamic and multi-functional nature of collagen. The interactome suggests stark differences in fibril structure between quiescent and active states, such as in the presence of tissue injury. These findings have important implications in regenerative medicine, including the generation of collagen mimetics with instructive cues such as angiogenic properties.

Another focus area of is the application of collagen towards various aspects of biomedicine, including wound healing, airway mechanics, cancer, and cardiovascular diseases. Mathew-Steiner et al. overview the role of collagen in the regulation of skin wound healing. As an important component of the skin, collagen modulates multiple aspects of wound healing, including inflammation, angiogenesis, and ECM remodeling [31]. Collagen has been tested as an adjunctive therapy to promote wound healing in clinical trials, and this article provides a review of the state of collagen-based products for use in wound care. Another strategy for wound healing management is treatment with decellularized collagen-rich tissues derived from the placenta, dermis, small intestine submucosa, and other sources. Yeung et al. describe that the application of decellularized tissues has been tested for the treatment of burns, wounds, soft tissue repair, and reconstructive surgery [32]. The authors further describe the recent development of non-decellularized allografts that further support tissue repair through the activity of the transplanted cells. Additionally, experimental strategies to support wound healing include the formation of microvascular networks. In comparison to collagen hydrogels, fibronectin-modified collagen hydrogels were shown to support more vascularization of co-cultured cells composed of human umbilical vein endothelial cells and human-induced pluripotent stem-cell-derived smooth muscle cells. Duan et al. further demonstrated that α_v_β_3_ integrin signaling played an important role in supporting vascularization within the hydrogels [33]. These papers illustrate the importance of collagen as a modulator of wound healing processes.

In the area of airway mechanics, collagen is a major element of the airway components, including the larynx, trachea, bronchi, and bronchioles. Liu et al. discuss the current knowledge on collagen’s role in airway diseases and associated stiffness changes. The authors discuss how changes in collagen deposition, composition, crosslinking, and the resulting mechanical changes are associated with diseases such as cystic fibrosis, asthma, and chronic obstructive pulmonary disease [34]. Understanding collagen remodeling will provide new avenues to treat these diseases by modulating airway mechanics.

The spatial organization of collagen within tumors is an area of research that is highly clinically relevant. Ouellette et al. review the field of collagen fiber patterning and its association with tumor types and stages of disease [35]. The authors discuss the potential of collagen fiber patterning as a cancer biomarker or prognostic indicator. An additional area of application is the small angle X-ray scattering (SAXS) as a cancer diagnostic tool sensing the local collagen organization [36,37,38,39]. The direct correlation between 3D collagen fibril orientation and its SAXS was recently demonstrated [40]. The areas lacking in fundamental knowledge are also described, including the mechanistic insight into how and why collagen undergoes spatial patterning within tumors.

Sapudom et al. systematically study the infiltration and phenotypic modulation of a monocytic cell line as a function of collagen fibril density [41]. The authors show that dense collagen matrices induced an upregulation in macrophage-specific gene expression, as well as the secretion of inflammatory-related cytokines. In the area of adipogenesis, Newman et al. studied the role of 3D hybrid hydrogels composed of collagen and elastin-like peptides in inducing adipogenesis of adipose-derived stem cells [42]. The authors show that the biochemical composition of the scaffold, the degree of crosslinking, and the mechanical properties all modulated adipogenesis. The authors report that stiff and/or crosslinked hybrid hydrogels conferred spheroid-like cellular organization that contributed to adipogenesis by fibronectin-functionalized collagen. Regarding applications in cardiovascular medicine, inducing the synthesis of elastin and collagen to the aortic vessel wall may be a therapeutic approach for the treatment of abdominal aortic aneurysm. Cunnane et al. assessed the role of extracellular vesicles derived from adipose stromal cells on smooth muscle cell ECM remodeling [43]. The authors reported that the extracellular vesicles promoted smooth muscle proliferation, migration, and the deposition of collagen and elastin. These results suggest that extracellular vesicles may be a potent modulator of ECM protein synthesis in smooth muscle cells that may have translational implications for the treatment of abdominal aortic aneurysms or for vascular tissue engineering applications.

In conclusion, collagen is highly abundant in the body, and its abnormal deposition or processing is associated with numerous diseases. This collection highlights the advances and opportunities for collagen to advance fundamental knowledge of matrix biology, as well as to apply them to the treatment of a variety of diseases and injuries. Collagen is actively being studied in a variety of diseases at preclinical and even clinical stages. In the future, we anticipate that the convergence of technological advances in computational biology and personalized medicine will enable a deeper understanding of how collagen can be targeted for regenerative medicine applications. For these reasons, we believe that collagen will remain a highly active area of research in the biomedical community in the decades to come. 
